# Deficiency of IFNAR1 Increases the Production of Influenza Vaccine Viruses in MDCK Cells

**DOI:** 10.3390/v17081097

**Published:** 2025-08-08

**Authors:** Qi Wang, Tuanjie Chen, Mengru Feng, Mei Zheng, Feixia Gao, Chenchen Qiu, Jian Luo, Xiuling Li

**Affiliations:** Department of Virus and Vaccine, Shanghai Institute of Biological Products, Shanghai 200052, China

**Keywords:** IFNAR1, influenza vaccine, MDCK cells, interferon, CRISPR/Cas9

## Abstract

Cell culture-based influenza vaccines exhibit comparable safety and immunogenicity to traditional egg-based vaccines. However, improving viral yield remains a key challenge in optimizing cell culture-based production systems. Madin–Darby canine kidney (MDCK) cells, the predominant cell line for influenza vaccine production, inherently activate interferon (IFN)-mediated antiviral defenses that restrict viral replication. To overcome this limitation, we employed CRISPR/Cas9 gene-editing technology to generate an IFN alpha/beta receptor subunit 1 (IFNAR1)-knockout (KO) adherent MDCK cell line. Viral titer analysis demonstrated significant enhancements in the yield of multiple vaccine strains (H1N1, H3N2, and type B) in IFNAR1-KO cells compared to wild-type (WT) cells. Transcriptomic profiling revealed marked downregulation of key interferon-stimulated genes (ISGs)—including *OAS*, *MX2*, and *ISG15*—within the IFNAR1-KO cells, indicating a persistent suppression of antiviral responses that established a more permissive microenvironment for influenza virus replication. Collectively, the engineered IFNAR1-KO cell line provides a valuable tool for influenza virus research and a promising strategy for optimizing large-scale MDCK cell cultures to enhance vaccine production efficiency.

## 1. Introduction

Influenza is an acute respiratory disease caused by the influenza virus, resulting in seasonal epidemics and unpredictable pandemics that pose significant threats to both human and animal health [1,2,3,4,5,6]. To date, vaccination remains the most effective public health strategy for preventing viral transmission and reducing disease severity [7]. Although the traditional chicken-embryo-cultured influenza vaccine offers advantages such as mature technology, relatively low production costs, and scalable manufacturing capacity, it has several limitations: a production cycle of up to 26 weeks, the potential presence of egg-derived allergens, and adaptive mutations acquired by the virus during propagation in eggs, all of which may weaken the efficacy and safety of the vaccine [8,9,10,11,12].

In response to these challenges, mammalian cell culture technology has demonstrated significant advantages over the traditional chicken embryo-based production process, emerging as an innovative trend in influenza virus manufacturing. Among various cell substrates, Madin—Darby canine kidney (MDCK) cells have become the predominant cell line in influenza vaccine manufacturing due to their high viral susceptibility and robust growth characteristics [13,14,15]. The MDCK cell platform offers several advantages, including improved biosafety, greater process flexibility, and reduced risk of egg-related allergenic contaminants [16,17]. Accumulating evidence has demonstrated that MDCK-derived vaccines exhibit strong immunogenicity and protective efficacy [15,16,18,19]. Nevertheless, one persistent challenge is achieving viral yields comparable to those obtained from traditional egg-based methods. Therefore, enhancing viral replication efficiency in MDCK cells is critical for the broader adoption and scalability of this production system.

As the primary defense system, the host’s innate immune system activates a complicated antiviral response upon influenza virus infection [20,21,22]. Viral RNA recognition by cytosolic pattern recognition receptors (PRRs) triggers type I interferon (IFN-I) production, which subsequently initiates a biphasic signaling cascade through high-affinity binding to the interferon α/β receptor subunit 1 (IFNAR1) [23,24]. This interaction activates the JAK-STAT signal transduction cascade, resulting in the transcriptional upregulation of over 300 interferon-stimulated genes (ISGs) that establish an antiviral state [25,26]. Previous studies have demonstrated that genetic modification of key components in the IFN signaling pathway can enhance viral yield in MDCK cells. For instance, knockout (KO) of IRF7 has been shown to improve influenza virus replication [27]. Moreover, the American Type Culture Collection (ATCC)-developed STAT1-KO MDCK cell line (CCL-34-VHGTM) exhibits enhanced permissiveness to viral infection, highlighting the potential of targeting upstream regulators in the IFN signaling cascade. Therefore, systematic KO of components in the IFN signaling pathway, particularly upstream regulators such as IFNAR1 that occupy a hierarchical position above STAT1, may offer a promising approach for enhancing influenza virus production.

In this study, we generated an IFNAR1-KO adherent MDCK cell line using CRISPR/Cas9 gene-editing technology. Comparative analyses of viral replication kinetics across multiple influenza vaccine viruses of different subtypes were performed between WT and IFNAR1-KO cells. These results not only elucidate IFNAR1’s critical function in antiviral immunity but also advance our understanding of IFN signaling dynamics during viral infection.

## 2. Materials and Methods

### 2.1. Cells and Virus

Wild-type (WT) MDCK cells (CCL-34, ATCC) were grown in Dulbecco’s Modified Eagle’s Medium (DMEM) (Gibco, Carlsbad, CA, USA), containing 10% fetal bovine serum (FBS) (Gibco, Carlsbad, CA, USA) at 37 °C and 5% CO_2_. The influenza vaccine strains used in this study included A/Victoria/2570/2019 (IVR-215, H1N1), A/Darwin/9/2021 (SAN-010, H3N2), and B/Austria/1359417/2021 (BVR-26, B Victoria Lineage) and were stored at −80 °C in our laboratory.

### 2.2. IFNAR1 Knockdown in MDCK Cells

Small interfering RNAs (siRNAs) targeting the canine IFNAR1 (siIFNAR1) and a non-targeting negative control (siNC) were designed and synthesized by Sangon Biotech (Shanghai, China). The sense and antisense sequences of siIFNAR1 were 5′-CCGAAGAUAAGACAAUCAUTT-3′ and 5′-AUGAUUGUCUUAUCUUCGGTT-3′. The sense and antisense sequences of siNC were 5′-UUCUCCGAACGUGUCACGUTT-3′, and 5′-ACGUGACACGUUCGGAGAATT-3′. The synthetic siIFNAR1 or siNC was transfected into MDCK cells using Lipofectamine RNAi MAX (Invitrogen, Carlsbad, CA, USA). Forty-eight hours post-transfection, cells were harvest and lysed using Trizol (Invitrogen, Carlsbad, CA, USA) for RNA extraction.

For viral infection experiments, MDCK cells were transfected with siIFNAR1 or siNC for 24 h and then infected with the IVR-215 virus at a multiplicity of infection (MOI) of 0.001. The infection medium consisted of DMEM supplemented with 2 μg/mL N-tosyl-L-phenylalanine chloromethyl ketone (TPCK)-treated trypsin (Sigma-Aldrich, Saint Louis, MO, USA). Following 48 h of virus incubation, cells and supernatant were collected for RNA extraction and viral titration separately.

### 2.3. RNA Extraction and Reverse Transcription

Total RNA was isolated from each sample using TRIzol^®^ reagent. Subsequently, 1 μg of purified RNA was reverse-transcribed into complementary DNA (cDNA) using a cDNA Synthesis Kit (Vazyme Biotech, Nanjing, China).

### 2.4. Quantitative Real-Time PCR (qPCR)

qPCR analysis was carried out using ChamQ Universal SYBR qPCR Master Mix (Vazyme Biotech, Nanjing, China). All reactions were conducted in triplicate. Canine glyceraldehyde-3-phosphate dehydrogenase (GAPDH) was used as the endogenous reference gene for normalization. Primer sequences used for amplification are listed in Table 1 [28].

### 2.5. Construction of Plasmids Targeting Canine IFNAR1

To generate a guide RNA (gRNA) targeting the canine *IFNAR1* gene, we used the CRISPR design tool available at https://www.zlab.bio/ (accessed on 11 March 2023) to identify a high-scoring sgRNA sequence. The chosen sgRNA, located on the third exon of the *IFNAR1* gene (accession number: NC_051835.1), is 5′-AAGGAAACAACACTTCTCCG-3′. Both the forward and reverse primers (as detailed in Appendix A Table A1) incorporated a BbsI restriction site and were successfully annealed. The DNA template was subsequently inserted into the PX459 vector (GenScript, Nanjing, China) and transformed into the *E. coli* competent cell strain DH5α (Takara, Dalian, China). The recombinant plasmid, designated PX459-IFNAR1, was verified through Sanger sequencing and subsequently purified using the Maxi Plasmid Kit (Tiangen, Beijing, China).

### 2.6. Generation of IFNAR1-KO Cell Lines

To achieve optimal transfection efficiency in MDCK cells, a mixture of 4 μg of the PX459-IFNAR1 plasmid and Lipofectamine 3000 (Invitrogen, Carlsbad, CA, USA) was co-incubated with 7 × 10^5^ MDCK cells. Following a 10 min incubation at 37 °C, the transfection mixture was transferred to a 6-well plate and cultured for 48 h. Subsequently, puromycin (4.5 μg/mL) was added to the medium. After 7−10 days of selection, monoclonal cell populations were isolated from surviving colonies via limited dilution.

### 2.7. PCR and Sequencing

Genomic DNAs from candidate monoclonal cell lines were extracted using a DNA Isolation Kit (Vazyme Biotech, Nanjing, China). Specific primers (listed in Appendix A Table A2) were designed to amplify the targeted region of IFNAR1. The PCR product was confirmed using 1% agarose gel and subsequently sent to Sangon Biotech (Shanghai, China) for sequencing.

### 2.8. Cell Density Curve Assay

WT and IFNAR1-KO MDCK cells were seeded into 24-well plates at an initial density of 1 × 10^4^ cells/well. Cells were harvested every 24 h up to 168 h post-seeding. At each time point, cells were detached using Trypsin-EDTA (Gibco, USA), centrifuged at 1000rpm for 5 min, and resuspended in 1 mL of DMEM. A 10 μL aliquot of the cell suspension was mixed with an equal volume of acridine orange/propidium iodide (AO/PI) staining solution (Nexcelom, Lawrence, MA, USA) and analyzed using the Cellometer^TM^ K2 instrument (Nexcelom, Lawrence, MA, USA) to determine cell concentration and viability. Cell growth curves were plotted based on viable cell counts over time.

### 2.9. Western Blot

Cell samples were lysed with cell lysis buffer (Beyotime, Shanghai, China) and incubated on ice for 30 min to ensure complete lysis. Lysates were then cleared by centrifugation at 10,000× *g* for 5 min at 4 °C. Supernatants were collected, mixed with loading buffer, and boiled for 10 min. Proteins were separated by SDS-PAGE and transferred onto a PVDF membrane (Millipore, Bedford, MA, USA). The membrane was blocked with 5% non-fat milk in TBST for 1 h and incubated with primary antibodies diluted in 5% non-fat milk/TBST (1:1000 dilution) overnight at 4 °C. The primary antibodies for detection included IFNAR1(ABclonal, Wuhan, China), β-actin (Transgene, Beijing, China), NP (Abcam, Cambridge, UK), and GAPDH (Genscript, Nanjing, China). After three washes with TBST, membranes were incubated with HRP-conjugated secondary antibodies for 1h at room temperature. Protein signals were detected using an Azure Imaging System (Azure Biosystem, Dublin, CA, USA).

### 2.10. Determination of Virus Titer

MDCK cells were seeded into 96-well plates (2 × 10^4^ cells per well) before viral infection. Following incubation, the culture medium was replaced with 10-fold diluted samples for 1 h at 37 °C to allow viral adsorption. Subsequently, cells were washed, and fresh DMEM supplemented with 2 μg/mL TPCK-trypsin was added. The plates were incubated at 35 °C for 72 h. Then 50 μL aliquots of the cell culture supernatants were transferred to U-bottom 96-well microplates. Each aliquot was mixed with an equal volume (50 μL) of a 1% suspension of chicken red blood cells and incubated at room temperature for 30 min. HA titers were recorded and expressed as log_10_ HA units per 50 μL (log_10_ HAU/50 μL) [29]. TCID_50_ was calculated using the Reed–Muench method [30].

### 2.11. RNA-Seq and Data Analysis

WT and KO cells were washed with phosphate-buffered saline (PBS) (Sparkjade, Jinan, China) and lysed in TRIzol reagent at a density of 5 × 10^6^ cells per mL. Cell lysates were flash-frozen in liquid nitrogen and then submitted to Zhongke New Life Biotechnology Co., Ltd. (Shanghai, China), for library preparation and high-throughput RNA sequencing.

Raw sequencing reads were processed to remove adapter sequences and low-quality bases using standard quality control pipelines. Clean reads were aligned to the Canis lupus familiaris reference genome (Ensembl CanFam3.1) using HISAT2 (v2.2.0) [31]. Differentially expressed genes (DEGs) were identified using the following the criteria: |log2fold change| > 1 and *p* < 0.05. To characterize transcriptional divergence between WT and KO cells, multi-dimensional analyses were conducted [28,32,33].

### 2.12. Statistical Analysis

All statistical analyses were carried out using Student’s *t*-test. Each experiment included three biological replicates, and a *p*-value < 0.05 was considered significant.

## 3. Results

### 3.1. Knockdown of IFNAR1 Enhances Influenza Virus Replication in MDCK Cells

**Figure 1:**
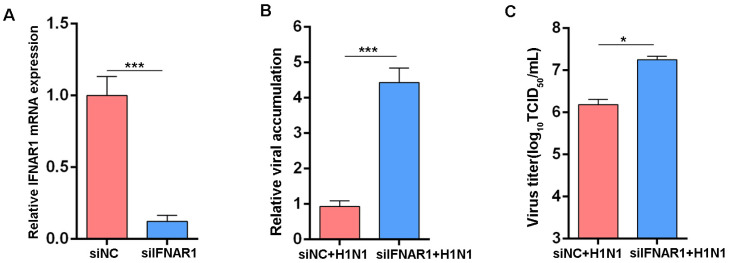
Effects of IFNAR1 knockdown on IAV replication. (**A**) RT-qPCR showing IFNAR1 knockdown by siRNA. (**B**) Relative levels of NP expression in cells. (**C**) Virus titers in supernatants. Canine *GAPDH* was used as the reference gene. * *p* < 0.05, *** *p* < 0.001 (Student’s *t*-test).

The IFNAR1 gene, a critical subunit of the IFN-I receptor complex. To elucidate its antiviral function in restricting influenza virus replication in MDCK cells, we performed RNA interference experiments using specific siRNAs targeting IFNAR1. RT-qPCR analysis confirmed a significant reduction in IFNAR1 mRNA expression in cells transfected with siIFNAR1 compared to those treated with the negative control siRNA (siNC) (Figure 1A). Following viral infection with an H1N1 (IVR-215) strain, IFNAR1-knockdown cells exhibited markedly higher levels of viral RNA accumulation and increased viral titers at 48 h post-infection (hpi), as shown in Figure 1B,C. These results indicate that IFNAR1 suppresses influenza virus replication in MDCK cells.

### 3.2. Generation of IFNAR1-KO MDCK Cell Line

**Figure 2:**
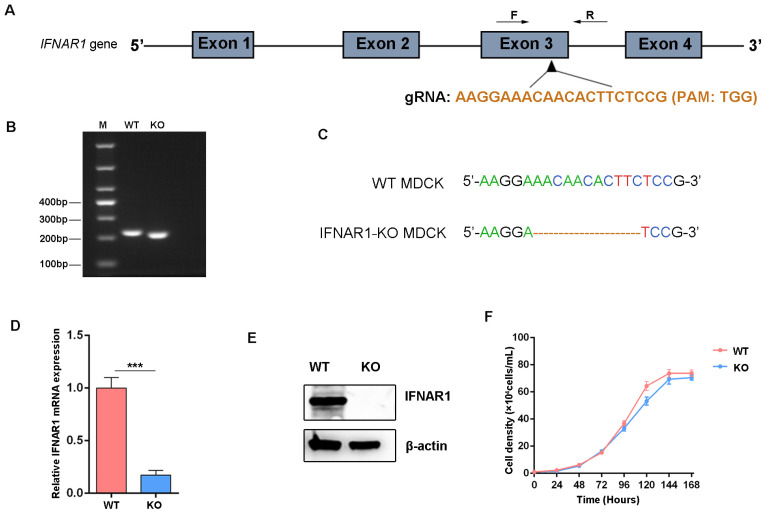
Construction of IFNAR1-KO MDCK cell lines. (**A**) Schematic map of the gRNA target sites on the genomic regions of IFNAR1. (**B**) Agarose gel electrophoretic analysis of genomic DNA fragmentation in WT and IFNAR1-KO cells. (**C**) Sequence alignment analysis results. (**D**) Relative transcription levels of IFNAR1 in WT and IFNAR1-KO cells. Canine GAPDH was used as the reference gene. *** *p* < 0.001 (Student’s *t*-test). (**E**) Western blot detection of IFNAR1 protein. (**F**) Cell density curves of WT and IFNAR1-KO cells.

To establish a stable IFNAR1-KO MDCK cell line, a guide RNA (gRNA) targeting the third exon of the IFNAR1 gene was designed and cloned into the PX459 vector (Figure 2A). Two days after transfection, puromycin was applied to select for cells expressing the CRISPR/Cas9 construct. Single-cell clones were then isolated via limiting dilution for further analysis. Genomic DNA was extracted from individual clones, and PCR amplification spanning the target site was performed (Figure 2B). A 11 bp deletion at the cleavage site was identified through the sequence alignment of PCR products (Figure 2C).

To evaluate the KO efficiency of IFNAR1, both mRNA and protein expression levels were assessed (Figure 2D,E). The results showed a significant reduction in IFNAR1 expression at both the transcriptional and translational levels in the KO clone. In addition, cell growth kinetics were analyzed to assess the impact of IFNAR1 deletion on cellular proliferation. No significant differences were observed between WT and IFNAR1-KO MDCK cells over a 5-day period (Figure 2F), indicating that the KO did not compromise cell viability or growth capacity. These results demonstrate that IFNAR1-KO cells were successfully generated and retained normal growth behavior comparable to WT cells.

### 3.3. Transcriptomic Analysis of KO vs. WT Cells

**Figure 3:**
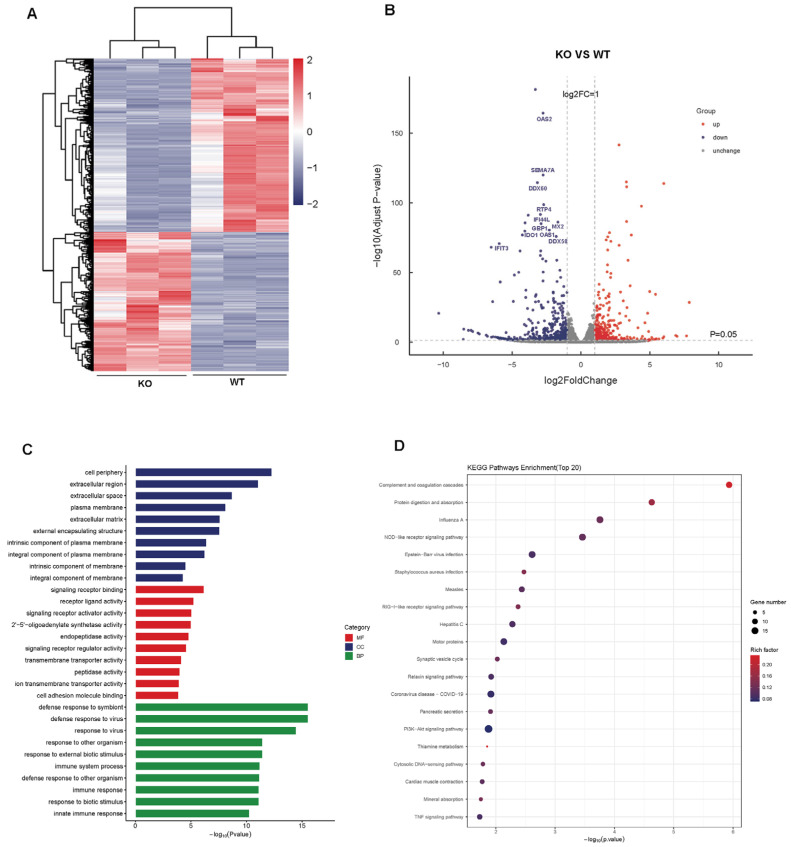
Transcriptome analysis of IFNAR1-KO vs. WT cells. (**A**) Heatmap of DEGs between IFNAR1-KO and WT cells. (**B**) Volcano plot shows DEGs between IFNAR1-KO and WT cells. (**C**) GO analysis of DEGs between IFNAR1-KO and WT cells. (**D**) Top 20 KEGG pathway analyses of DEGs between IFNAR1-KO and WT cells.

High-throughput RNA sequencing was performed to further characterize the IFNAR1-KO cells. A bidirectional hierarchical clustering heatmap of the expression levels of differentially expressed genes (DEGs) clearly separated IFNAR1-KO and WT samples into two distinct clusters (Figure 3A), confirming consistent transcriptional differences between the groups. The volcano plot showed that a total of 1064 DEGS were obtained, comprising 591 upregulated and 473 downregulated genes (Figure 3B). Notably, among the downregulated genes, there was a significant enrichment of interferon (IFN)-related genes, including OAS2, DDX58 (RIG-I), and IFIT3, which indicated that knockdown of IFNAR1 influence ISGs expression.

The Gene Ontology (GO) analysis encompasses three categories—biological processes, cellular components, and molecular functions—to elucidate the principal biological roles of these DEGs. The top 30 enriched GO terms are summarized in Figure 3C. In the biological process category, the DEGs are predominantly associated with immune-related activities, including defense response to virus (GO:0051607) and innate immune response (GO:0045087), underscoring the role of IFNAR1 in maintaining an antiviral state. Within molecular functions, terms such as the 2′–5′-oligoadenylate synthetase activity (GO: 0001730) and signaling receptor regulator activity (GO:0030545) were notably enriched, indicating functional alterations in the interferon signaling axis in IFNAR1-KO cells.

Pathway-level analysis using the Kyoto Encyclopedia of Genes and Genomes (KEGG) revealed that the top 20 downregulated pathways in IFNAR1-KO cells were predominantly associated with host–virus interactions (Figure 3D). Particularly, pathways related to influenza A virus infection (cfa05164) and RIG-I-like receptor signaling (cfa04622) were significantly suppressed. Overall, these findings suggest that a lack of IFNAR1 significantly decreases the inherent antiviral ability of MDCK cells, potentially facilitating increased viral replication.

### 3.4. Reduced Expression of IFN-Related Genes in IFNAR1-KO Cells

**Figure 4:**
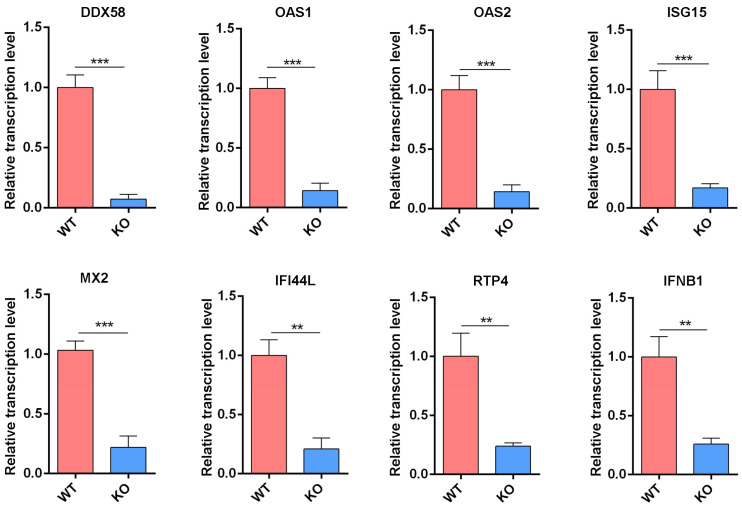
Transcription level of IFN-related genes verified by RT-qPCR. Total RNAs from WT and KO cells were extracted and RT-qPCR was carried out to examine the expression of genes including *DDX58*, *OAS1*, *OAS2*, *ISG15*, *MX2*, *IFI44L*, *RTP4* and *IFNB1*. The experiments were repeated three times independently. The mRNA level of WT cells was set as 1. Canine *GAPDH* was used as the reference gene. ** *p* < 0.01, *** *p* < 0.001 (Student’s *t*-test).

RT-qPCR analysis was conducted on key genes involved in the IFN-I signaling pathway to validate the transcriptome findings. Candidate genes were selected based on their functional relevance to IFN production and antiviral activity. Specifically, genes associated with IFN induction, such as *DDX58* (RIG-I) and *IFNB1* (IFN-β), were analyzed, along with those implicated in the effector phase of the antiviral response, including *OAS1*, *OAS2*, *ISG15*, *MX2*, *IFI44L*, and *RTP4*. The results showed a significant decrease in the mRNA expression levels of all selected genes in IFNAR1-KO cells compared to WT cells (Figure 4), consistent with the RNA-seq data. Consequently, these findings support the conclusion that IFNAR1 deficiency leads to impaired activation of IFN-related signaling pathways, thereby attenuating the intrinsic antiviral defense capacity of MDCK cells.

### 3.5. Increased Viral Titers in IFNAR1-KO Cells

**Figure 5:**
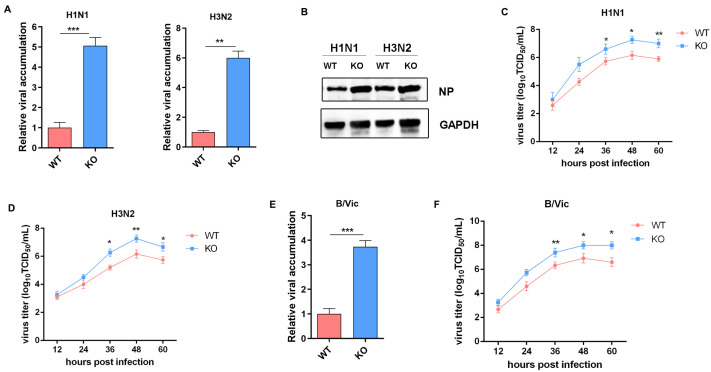
Comparison of the replication of different virus subtypes in WT and IFNAR1-KO cells. (**A**) QPCR detection of NP mRNA levels of H1N1 and H3N2 in different cells. (**B**) Western blot analysis of NP expression. (**C**) Viral titers of H1N1 in different cells. (**D**) Viral titers of H3N2 in different cells. (**E**) qPCR detection of NP mRNA levels of B/Vic in different cells. (**F**) Viral titers of B/vic in different cells. * *p* < 0.05, ** *p* < 0.01, *** *p* < 0.001 (Student’s *t*-test).

We hypothesized that the weakened antiviral response increased viral production in IFNAR1-KO cells. WT and IFNAR1-KO MDCK cells were infected with representative strains of influenza A (H1N1 and H3N2) and influenza B (B/Victoria). RT-qPCR results revealed significantly higher viral RNA accumulation in IFNAR1-KO cells compared to WT cells for both H1N1 and H3N2 subtypes (Figure 5A). Consistent with these findings, Western blot analysis showed markedly elevated NP protein expression in IFNAR1-KO cells (Figure 5B), confirming enhanced viral protein synthesis in the absence of functional IFNAR1 signaling. To comprehensively assess viral replication kinetics, time-course experiments were conducted following H1N1 infection. As shown in Figure 5C, viral titers increased in a time-dependent manner in both cell types. Notably, at 36 hpi, the viral titer in IFNAR1-KO cells was significantly higher than that in WT cells. By 48 hpi, the titer in KO cells exceeded 1 × 10^7^ TCID_50_/mL—more than three-fold higher than that in WT cells. Similar trends were observed in H3N2-infected cells, where IFNAR1-KO cells exhibited a 3.9-fold increase in viral titer compared to WT cells at 36 hpi.

Infections with the B/Victoria strain also demonstrated enhanced replication in IFNAR1-KO cells. At 48 hpi, qPCR revealed a 5.2-fold increase in viral load in IFNAR1-KO cells relative to WT cells (Figure 5D). Furthermore, viral titration showed that IFNAR1-KO cells reached 8.7 × 10^6^ TCID_50_/mL at 36 hpi, significantly higher than the 2.1 × 10^6^ TCID_50_/mL observed in WT cells (Figure 5E). Collectively, these results demonstrate that disruption of IFNAR1-mediated signaling substantially impairs the host antiviral response, leading to enhanced replication of multiple influenza virus subtypes, including both influenza A and B lineages.

### 3.6. Transcriptomic Analysis of IFNAR1-KO vs. WT Cells Following Viral Infection

**Figure 6:**
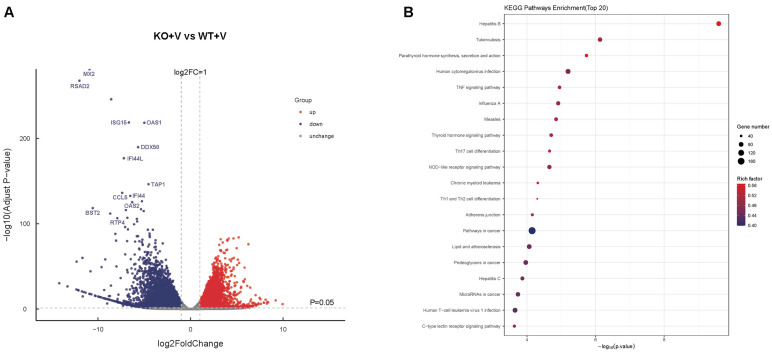
Transcriptome analysis of IFNAR1-KO vs. WT cells after viral infection. (**A**) Volcano plot shows DEGs between WT and IFNAR1-KO cells after viral infection. (**B**) Top 20 KEGG pathway analyses of DEGs between WT and IFNAR1-KO cells after viral infection.

To further explore the molecular differences between WT and IFNAR1-KO MDCK cells in response to viral infection, we performed high-throughput RNA sequencing following H1N1 influenza virus infection. Analysis revealed a total of 8242 DEGs, with 3663 genes upregulated and 4579 downregulated in KO cells compared to WT controls (Figure 6A). Notably, gene ontology and pathway enrichment analyses highlighted that a significant proportion of the downregulated DEGs were associated with IFN-I signaling pathways. Key antiviral effectors such as *MX2*, *RSAD2* (viperin), *ISG15*, and *DDX58* (RIG-I) were markedly reduced in expression in IFNAR1-KO cells.

Importantly, these critical genes exhibited similar downregulation patterns in uninfected WT and KO cells (Figure 3B), indicating that IFNAR1 KO exerts a pronounced suppressive effect on downstream ISG expression. KEGG pathway analysis identified the top 20 downregulated pathways in KO cells post-infection (Figure 6B). Among them, the “Influenza A” pathway was significantly suppressed, consistent with the enhanced viral replication observed in IFNAR1-KO cells. These findings suggest the critical role of IFNAR1 in orchestrating the host antiviral response upon influenza virus infection.

### 3.7. Validation of Gene Expression Following Viral Infection

**Figure 7:**
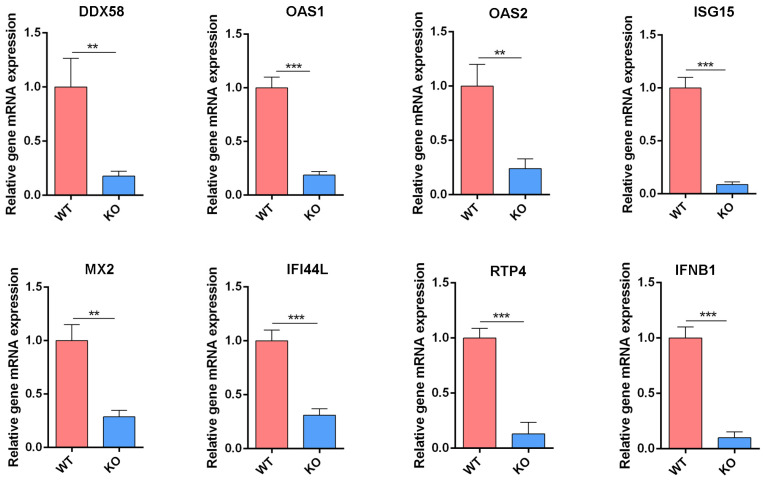
Transcription level of IFN-related genes verified by RT-qPCR. Total RNAs from WT and KO cells after viral infection were extracted and RT-qPCR was carried out to examine the expression of IFN-related genes. The experiments were repeated three times independently. The mRNA level of WT cells was set as 1. Canine *GAPDH* was used as the reference gene. ** *p* < 0.01, *** *p* < 0.001 (Student’s *t*-test).

To further validate the downregulation of key IFN-related genes identified in the transcriptomic analysis, we assessed the mRNA expression levels of selected antiviral genes in WT and IFNAR1-KO cells following H1N1 virus infection. As shown in Figure 7, the induction levels of antiviral genes—including *DDX58*, *OAS1*, *OAS2*, *ISG15*, *MX2*, *IFI44L*, *RTP4*, and *IFNB1*—were markedly reduced in KO cells compared to WT cells post-infection. These results corroborate the RNA-seq data and support the hypothesis that the enhanced viral replication observed in IFNAR1-KO cells is closely associated with the impaired activation of downstream antiviral signaling pathways due to the loss of IFNAR1-mediated signaling.

## 4. Discussion

Cell culture-based influenza vaccines have emerged as a superior alternative to traditional egg-based production systems, positioning themselves at the forefront of next-generation vaccine development [8,34]. However, scaling up cell-based influenza vaccine production remains challenging, with the enhancement of viral titers representing a critical barrier. Key limiting factors include host cell antiviral responses, viral replication efficiency, and suboptimal culture conditions [35,36,37,38]. Among these, the innate antiviral immune response, particularly those mediated by IFN signaling, represents one of the most significant factors to inhibit efficient viral propagation.

To address this issue, recent studies have focused on engineering cell lines with impaired IFN signaling to enhance viral yield. For instance, STAT1-KO cell lines established by ATCC exhibit up to 30-fold higher titers of IAV compared to WT cells, without compromising genetic stability across passages—making them suitable for large-scale manufacturing. Similarly, IRF7-deficient MDCK cells demonstrate increased yields of both IAV and IBV, with virus titer enhancements of approximately 1.5- and 2-fold, respectively [27]. Establishment of the MAVS-inactivated MDCK cell line markedly improved the replication efficiency of canine distemper virus (CDV) [39]. Additionally, deletion of IFITM3 in human cells has been shown to enhance susceptibility to IAV infection [40], further supporting the notion that suppression of IFN-mediated antiviral defenses can promote viral replication.

In this study, we investigated the role of IFNAR1, a key subunit of the type I IFN receptor complex, in regulating antiviral immunity and influenza virus replication [41]. Upon binding of IFN-α or IFN-β, the IFNAR heterodimer (composed of IFNAR1 and IFNAR2) recruits the tyrosine kinases TYK2 and JAK1, initiating the JAK-STAT signaling cascade [25,42]. This pathway not only drives the transcription of ISGs but also regulates diverse cellular processes such as proliferation, apoptosis, differentiation, and immune activation [43,44]. Previous studies have shown that the deletion of IFNAR1 in Madin–Darby bovine kidney (MDBK) cells markedly promotes the replication efficiency of bovine virus [45]. Our initial experiments using siRNA-mediated knockdown of IFNAR1 in MDCK cells revealed a significant increase in viral replication, suggesting that IFNAR1 is a viable target for genetic modification aimed at improving viral yield.

Based on the above findings, we employed CRISPR/Cas9 technology to generate a stable IFNAR1-KO MDCK cell line. Growth kinetics analysis confirmed that the loss of IFNAR1 did not impair cellular proliferation or viability, which is essential for maintaining industrial scalability and batch consistency in vaccine manufacturing. Infection assays demonstrated that the viral titers of H1N1, H3N2, and B/Victoria vaccine strains were increased by 3.3-, 3.9-, and 4.1-fold, respectively, in IFNAR1-KO cells relative to WT cells, indicating that IFNAR1 deletion enhances the replication of various influenza virus subtypes commonly used in vaccine production. Notably, studies have reported that some H1N1 and H3N2 strains exhibit reduced replication efficiency in eggs, with viral yields frequently limited by strain-specific adaptation mutations [46,47]. The enhanced viral replication observed in IFNAR1-KO cells suggests that this engineered cell line may serve as a better substrate for propagating low-yield strains, potentially reducing the number of required amplification passages and shortening production timelines.

Transcriptomic profiling of IFNAR1-KO cells revealed widespread downregulation of ISGs, confirming the central role of IFNAR1 in IFN signal transduction. Similarly, previous studies have revealed significant differences in the antiviral IFN response between two adherent MDCK cell lines with distinct viral yields [35]. Our earlier work has also demonstrated that adaptation of MDCK cells to serum-free suspension culture is associated with reduced expression of ISGs, which in turn facilitates enhanced viral replication [28]. This observation further underscores the importance of modulating the IFN pathway to optimize vaccine production platforms. From a translational perspective, the IFNAR1-KO cell line serves dual purposes: (1) as a powerful tool for studying virus–host interactions, and (2) as a scalable platform for high-titer influenza vaccine production. In addition, this platform enables future genetic engineering of functionally relevant genes, such as those involved in tumorigenicity for improved cell safety, or further optimization of suspension MDCK cells to enhance viral production under scalable bioprocess conditions [38,48,49,50].

## 5. Conclusions

**Figure 8:**
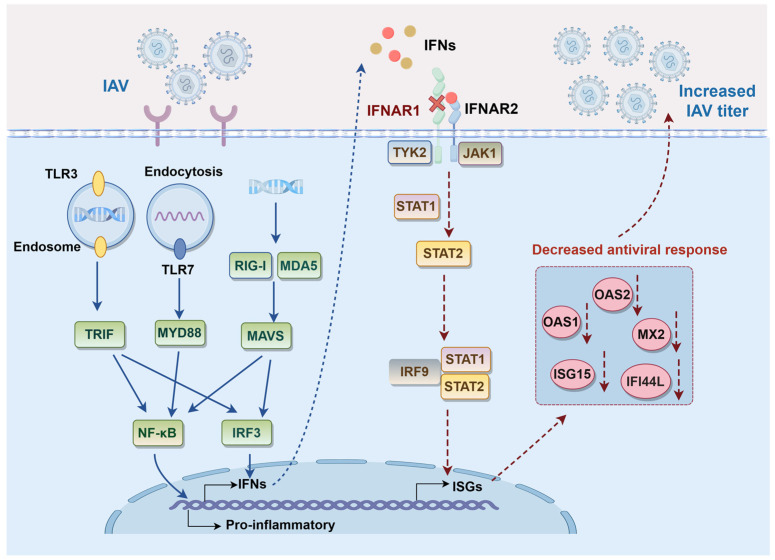
Schematic model of IFNAR1 KO to affect IAV replication in MDCK cells.

In conclusion, we constructed an IFNAR1-KO cell line that enhances the yield of various influenza virus strains, with the high productivity being related to the downregulation of the IFN response (Figure 8). These findings not only contribute to the mechanistic understanding of host antiviral responses but also provide a novel bioprocessing strategy for optimizing large-scale MDCK cell culture systems in influenza vaccine production.

## Figures and Tables

**Table 1:** Primers used for qPCR.

Primer Name	Sequence (5′-3′)
cIFNAR1 mRNA-F	GAGCAGAGGAAGGAAACAAC
cIFNAR1 mRNA-R	CAGGCAGGGAGATGTTTATTATG
cRIG-I mRNA-F	AGAGCAAGTTCAGTCAACTGTG
cRIG-I mRNA-R	ACTGAAGGTGGACATGGGTC
cOAS1 mRNA-F	TCTTCAGGCAAAGGCACGAC
cOAS1 mRNA-R	ACTCTGGACCTCAAAGTACAC
cOAS2 mRNA-F	GGTGAAGTATTGGTACCAACAGTG
cOAS2 mRNA-R	CTGCTCCTGCTGTCTGATTAG
cISG15 mRNA-F	CATGGCTGGGAACCTGACTG
cISG15 mRNA-R	GAGATCCCATCCTGCAGCAC
cMX2 mRNA-F	ACTTGGCATCAGCCATGAGC
cMX2 mRNA-R	TGATCGTCTCTTGCTTCTGGATG
cIFI44L mRNA-F	ACCAGCATTACTGAGCAGTATAG
cIFI44L mRNA-R	TGTAGGGTATGTCTTCCATGC
cRTP4 mRNA-F	CTGAGGTCACGAAACAACACAAC
cRTP4 mRNA-R	TGGGTCTCAGTAGTGGTATGGC
cIFNβ mRNA-F	GAGCAACGACTTGCTTCGATC
cIFNβ mRNA-F	CTGGAACTGGCGTGATTTCTC
cGAPDH mRNA-F	CCAAGAGGGTCATCATCTCTGC
cGAPDH mRNA-R	TGCCGAAGTGGTCATGGATG
IVR-215 NP-F	CCAAGCAAACAATGGCGAAG
IVR-215 NP-R	CTTTTACTGCAGCACCTGC
SAN-010 NP-F	GTGTGGATGGCATGCCATTC
SAN-010 NP-R	CTCCGCTCCTGGTCCTTATG
BVR-26 NP-F	CAGAGATAAAGAAGAGCGTCTAC
BVR-26 NP-R	TTCTTGTCATCAGTGGCAGC

## Data Availability

The data presented in this study are available on request from the corresponding author. The data are not publicly available due to security restrictions.

## Appendix A

**Table A1:** Primers used for annealing.

Primer Name	Sequence (5′-3′)
F1	CACCGAAGGAAACAACACTTCTCCG
R1	AAACCGGAGAAGTGTTGTTTCCTTC

**Table A2:** Primers used for amplifying the targeted region of IFNAR1.

Primer Name	Sequence (5′-3′)
F2	CTTGGTTAAAATTGCCTGGGTG
R2	GCCCTTATGGTAACAGTAAACGAG
